# Engaging children and adolescents in the design and conduct of paediatric research

**DOI:** 10.3389/fped.2024.1481754

**Published:** 2024-11-12

**Authors:** Barbara E. Bierer, Elisa Koppelman, Alysha K. Croker, Sharareh Hosseinzadeh, Collin Hovinga, Steven Joffe, Gianna McMillan, Robert Nelson, Christina Bucci-Rechtweg

**Affiliations:** ^1^ Division of Global Health Equity, Department of Medicine, Multi-Regional Clinical Trials Center of Brigham and Women’s Hospital and Harvard, Brigham and Women's Hospital, Boston, MA, United States; ^2^ Department of Medicine, Harvard Medical School and Brigham and Women’s Hospital, Boston, MA, United States; ^3^ Health Canada, Centre for Policy, Pediatrics and International Collaboration at Health Canada, Ottawa, ON, Canada; ^4^ Global Head Clinical Patient Engagement, Chief Medical Office, Novartis Pharma, Toronto, ON, Canada; ^5^ Critical Path Institute (C-Path), Tuscon, AZ, United States; ^6^ Department of Medical Ethics and Health Policy, Perelman School of Medicine, University of Pennsylvania, Philadelphia, PA, United States; ^7^ Independent Patient & Participant Advocate, Los Angeles, CA, United States; ^8^ Johnson & Johnson, Pediatric Drug Development, Spring House, PA, United States; ^9^ Maternal Health and Pediatric Regulatory Policy, Novartis Pharmaceuticals Corporation, East Hanover, NJ, United States

**Keywords:** paediatrics, patient perspective, patient engagement, product development, regulatory approval

## Abstract

The importance of patient engagement in product development and clinical research is widely acknowledged. In pediatrics, parents and guardians are often vocal advocates for their children in the process, but investigators and sponsors rarely directly solicit children's or adolescents’ perspectives in clinical research planning or as patient partners during the conduct of research. Here, we provide compelling reasons and recommendations for investigators and sponsors to systematically engage young people in the design, conduct, and review of research, and the premise that input will be incorporated as a routine expectation. We consider the theoretical, ethical, and practical implications of this approach.

## 1 Introduction

The development, testing, authorization, and administration of safe and effective therapeutic products, including child-specific products, are critical to the health of populations. Younger children are affected differently than adults by diseases; they metabolize medications and respond to treatment differently than adults (1, 2). Further, children and adolescents may be impacted clinically and emotionally differently than adults, even if the response to the disease and medications are similar. They have perspectives, feelings, motivations, and priorities that might differ from adults (or adult parents of involved children) that may impact multiple aspects of clinical research. Other important differences in the design and conduct of research with children include the need for parental or guardian permission for participation and, where appropriate, for the child's assent. Importantly, the term “children” inadequately describes the population: changing physical and psychological growth and evolving maturity present significant differences across the paediatric age spectrum. Children aged 10–17 may share more similarities with adults in both biology and decision-making capacity than with neonates, infants, and young children. We will use the ICH E11 terms “young children” (defined as the population aged 2–6 years), “older children” (defined as aged 7–11 years), “adolescents” (defined as 12–16 or 18 years, depending on geography), and “young people” as an umbrella term encompassing the spectrum of youth aged 2–18 years (2).

As with adult patients and advocates, parents and guardians increasingly engage with medicines development and clinical research enterprises, particularly in oncology and rare disease communities (3–5). However, despite the need for an older child or adolescent participant's assent in most settings, the perspectives of these individuals are rarely solicited directly in clinical research planning (6–8). Here we argue that individuals responsible for designing, conducting, reviewing, and approving paediatric research should systematically and routinely engage with (older) children and adolescents. Their perspectives are valuable and should be solicited and shared with decision-makers, protocol designers, institutional review boards (IRBs)/research ethics committees (RECs), and regulatory agencies. We consider the theoretical, ethical, and practical implications of this approach.

Understanding the lived experience of having a disease or condition is foundational to patient-centric research. Patients alone can speak to their individual needs and symptoms, the perceived benefits and challenges of treatment, and the cumulative impact of these elements on their lives. They can suggest priorities, study questions, study endpoints, and trial logistics. They can review study materials, suggest ways to minimize burden, provide advice on recruitment materials and strategies, and propose patient-reported outcomes (PROs). The value of such input—albeit by adults—has been demonstrated (9). Improved product development and/or post-market clinical research has been achieved through adult patient partner involvement (10–13). Understanding patient perspectives can inform eligibility criteria, increase recruitment through community communication, improve retention by decreasing participant burden, and reduce costly protocol amendments and study delays. Patients’ perspectives may also inform and justify the benefit-risk analysis in regulatory decision-making (14).

Many entities have programs to formalize patient engagement. The US Food and Drug Administration (FDA) developed the Patient-Focused Drug Development program to collect and utilize patient perspectives (1, 15, 16). The European Medicines Agency (EMA) piloted a process to involve patient organisations in benefit-risk evaluations and updated its engagement framework (17–21). The Patient-Centered Outcomes Research Institute requires patient partnership, beginning with research planning, through dissemination of results (22). The Canadian Institutes of Health Research established a national strategy for patient-oriented research and encourages its integration in Canadian health research (23). In Europe, Patient Preferences in Benefit-Risk Assessments during the Drug Life Cycle, a public-private research project, is developing recommendations on inclusion of patients’ perspectives into decision-making (24). Professional organizations have also committed to patient engagement (25). None of these initiatives expressly consider *paediatric* patients and participants input (17). A 2019 scoping review of studies that collected primary data from people aged 12–18 years found that, of 420 health studies identified, only 21 (5%) reported youth involvement in the research process (26).

Paediatric engagement in research processes does exist. Multiple organizations support young people advisory groups globally, with some effort dedicated to clinical trial experience (27–33). The Duchenne Muscular Dystrophy (DMD) rare disease community advocates for a patient treatment model that incorporates data on family perspectives, involving an international network of DMD families (34, 35). While roadmaps to guide adult patient and parent/guardian participation exist, parallel efforts with paediatric partners remain infrequent. The rare engagement of young people may relate to efforts to protect “vulnerable” populations, skepticism regarding the value of paediatric patient input, and/or practical challenges in reaching an informed population, among others. There is a case for change: paediatric patient and participant partners should routinely and systematically be directly engaged in the clinical trial process. Young people can be engaged to identify key issues important to their age group, evaluate study logistics in light of their lives, advise on acceptable levels of life disruption, engage in recruitment modalities, and gauge the readability and accessibility of written content in any medium.

## 2 Discussion

The meaningful engagement of paediatric patients and participants in study design and conduct requires consideration of ethical concerns, patient engagement methodology, issues of representativeness, and the roles and responsibilities of involved parties.

### 2.1 Ethical perspectives

Legally prohibited from independently providing consent, children—of all ages—are designated as a population in need of additional safeguards for research participation (36, 37). The protectionism and concern over vulnerability extend to considerations of paediatric patient partners in the research process. There may be concerns that soliciting paediatric input in study design and conduct may lead to unintended harm, including privacy concerns, psychological distress, or dignitary harm; these can be mitigated by confidentiality measures and sensitivity to the nature, form, and content of the engagement and planning discussions (see below). As demonstrated by youth advocacy organizations, young people want their voices heard in research and product development; such organizations can help provide the infrastructure, policies, and support to young people in the planning phases.^1^^,^^2^^,^^3^ As experience grows, the broader effort to systematize and embed the paediatric perspective in product development and the clinical research lifecycle should be iterative.

An additional ethical concern is whether serving as a paediatric patient partner changes (or should change) participation eligibility on a trial on which they have advised since that patient partner will have privileged knowledge about the trial. Bias and data integrity concerns over the inclusion of an individual as a trial participant who has prior knowledge of the trial through planning involvement can be mitigated. If knowledge of trial design, research procedures, or endpoints could impact data integrity, that should be delineated and explained in advance; the patient partner role should only be offered to those who decline participation or would be otherwise ineligible (e.g., disease stage, disease comorbidity). While exclusion from clinical trial eligibility may be important, an alternative posture might argue that a person who participated in the planning process should be prioritized for enrollment, given their volunteer involvement and service. If the trial has limited enrollment capacity or is anticipated to have competitive enrollment (e.g., a new intervention for an unmet medical need for a rare disease), methods of identifying potential participants should be determined and explained in advance. It must be made clear that one's role as a pediatric patient partner is not connected to potential eligibility or clinical study participation; agreeing to serve as a patient partner should neither advantage nor disadvantage a patient's eligibility or influence fair selection of trial participants.

Payment and compensation for clinical trial participation have often been considered a risk of undue inducement of participation (38). Merits of this position as applied to paediatric research participation aside, payment is not an ethical concern in the context of patient partners. Patient engagement in study design is simply an invitation to be heard; risks are minimal, and there may be significant benefits for the young person, who might then better appreciate their own impact and agency. Paediatric patients and participants, and involved parents/guardians, should be able to receive reimbursement for expenses incurred and potentially compensated for their time and expertise, if the latter is consistent with the local and regional practices for payment.

### 2.2 Informing paediatric patient-reported outcomes

Patient-reported outcomes (PROs) contribute timely, relevant information on clinical benefits and complement efficacy and safety data (39, 40). Engagement of and co-creation by paediatric advisors (and parents/guardians) should be integral to the development of child- and adolescent-specific PROs –with age-appropriate interactions that consider language and culture. Public investment in PRO development includes the validation of instruments intended for children, despite the variable involvement of children and families as partners (41–43). The positive impact and validity of incorporating the child's voice in PRO measure development has been empirically demonstrated through development of the Paediatric Patient-Reported Outcomes version of the Common Terminology Criteria for Adverse Events (44, 45).

Thus far, global PRO collaboration has focused on evaluating methodologies, representativeness, evidence generation, and the appropriate use of patient experience data, with particular attention paid to young people and parents/guardians. Initiatives such as the International Council for Harmonisation (ICH) guidelines on patient-focused drug development will increase understanding of the utility, limitations, and value of PROs for IRB/REC assessments and regulatory decision-making that will advance patient-centricity, equity, and public health (46).

### 2.3 Methodology of engagement with young people

As paediatric patients and research participants become more involved with study planning, empirical measures to evaluate methodologies for and the impact of their engagement must be developed. Paediatric patient engagement must be well-planned, with personnel trained to manage the engagement and interaction, logistical engagement processes developed, and financial resources to support the activity made available. Paediatric patient partners can be consulted throughout all phases of the clinical trial lifecycle and product development. Table 1 highlights the value of integrated youth perspectives. It is critical for all involved adults to properly prepare for the engagement, and to ensure that the youth and adult carers (as applicable) are also properly prepared (47). Study questions should be crafted to ensure that patient partners can communicate their lived experience of the disease, views on study objectives, design, and outcome measures, and perspectives on the burdens and benefits of participation. Patients’ perspectives should be diverse and representative of those impacted by the condition and disease population demographics. Research ethics committees and regulatory health authorities should address whether and what information would be most helpful to their own deliberations.

**Table 1:** Contribution opportunities for paediatric patients to the clinical trial process.

Stage in clinical research & product development lifecycle	Utility of Information gathered
• Prioritization of research	• Communicate patient experience and burden of specific disease(s). • Identify current options for and gaps in treatment. • Identify troublesome symptoms that affect quality of life. • Help to prioritize development efforts within a disease for novel agents across and within classes of products for a disease. • Address patient education needs.
• Preclinical through clinical trials	• Understand the disease burden, symptoms and impact on quality of life with special consideration to developmental age and stage of the pediatric patient. • Understand the endpoints/outcomes (primary and secondary) that are most important to pediatric patients and their carers. • Address gaps in treatment and current standard of care. • Illuminate indications for study (study questions). • Highlight formulation considerations (route of administration, palatability, acceptability) with particular attention to age of patient. • Identify issues of convenience for study participants (burden of administration, scheduling, methods).
• Protocol design	• Provide input into study design, risk/benefit assessment, and risk tolerance of patient population. • Identify study population and clarify eligibility criteria; highlight issues related to participant reaching age of majority while on study. • Determine protocol acceptability (e.g., use of placebo, access to ancillary products). • Weigh in on study duration. • Comment on frequency of data collection, burden of research procedures, location. • Evaluate recruitment methods and communication channels. • Ascertain readability and adherence to health literacy principles of patient education, recruitment and instructional materials, and informed assent/consent, including user testing. • Provide input into digital technologies, including user testing. • Offer input into hybrid and decentralized trial design.
• IRB/REC review	• Explain perception of risk tolerance of patient population and carers as appropriate (as related to age of youth) in relation to life experience. • Provide insights into benefit/risk assessment. • Offer commentary on input to protocol in advance of submission. • Provide insights into informed consent/assent documents, process and procedural instructions.
• Study conduct	• Advise sponsors and investigators on patient recruitment and retention. • Communicate in age-appropriate manner on merits of a study. • Offer input into adverse event evaluation. • Share perspectives on participant feedback and complaints. • Respond to investigator questions and concerns.
• Study completion	• Help interpret and communicate study results. • Facilitate user testing of plain language summary with consideration of age-appropriate language and format. • Assist in communication to involved communities with consideration of relevant affinity groups. • Identify future opportunities for study.

The approach and methodology of paediatric engagement will vary depending on the disease, stage, study question, and planned outcome data. An exploratory study, for example, that requires drawing an extra tube of blood following a clinically necessary phlebotomy may not require paediatric patient partners: the burden, discomfort, and emotional distress of a blood draw are well-known, with limited alterative options. This scenario differs significantly from that of a novel treatment for Type 2 diabetes that may well benefit from paediatric patient involvement throughout all phases of the product development lifecycle.

The apparent benefits and goals of involving paediatric patient partners in clinical research track to those already delineated in adult populations (48, 49). Essential differences relate to the age group, maturity, and legal status of the paediatric population. Prior to initiating the paediatric patient/participant interaction, the goals should be well-defined and presented first to the parent/guardian whose permission must be secured in advance of approaching the young person. The research team should be prepared to discuss with the parent/guardian not only the purpose, nature, and content focus of the engagement, but also logistical and operational details. Engagement details must be determined, including the selection process for paediatric patient partners and their parent/guardian; communication about the consultation process; whether and how the participant(s) will be compensated; duration of the commitment; confidentiality provisions and assurances; logistical considerations such as meeting format (e.g., virtual or in-person interaction, individual or focus group discussion, alone or with the parent/guardian); data collection method(s); and provision of feedback to those patient partners. Whether and how the parent/guardian will be involved in future interactions should be discussed. The conversation with the parent/guardian must be understandable and sufficiently complete such that the parent/guardian can give free, informed consent for the researchers to approach the pediatric patient partner directly.

The concept of *partnership* with paediatric advisors (“paediatric partners”) should ground all interactions, with opportunities for feedback and modification. Systematic planning of paediatric engagement begins by assessing what is already known, identifying existing gaps in paediatric perspectives, and developing the scope of questions and issues for which the research team seeks input. Table 2, not intended to be prescriptive or exhaustive, offers exemplary questions and considerations to highlight preparing for engagement, where the youth perspective may clarify the nature and impact of the disease, barriers to trial participation, and patient and parent/guardian perspectives. Written materials related to participation should be age-appropriate, linguistically and culturally considerate, and follow health literacy principles.

**Table 2:** Systematic preparation for paediatric participant engagement.

Stages at which to prepare for specific types of patient engagement	Questions/considerations
Pre-engagement	• Set the stage for respectful engagement by attending to space, voice, audience and influence, as laid out in the Lundy Model which supports a child's right to participate, express views, and be heard. • Ensure the appropriate consent and assent processes have been completed with the requested activity thoroughly explained • Allow for ample time to engage with the selected youth (and carers). • Prepare for each engagement activity so setting, approach, word choice, materials and etc.is specifically and age-appropriately geared towards the youth. • Determine what is known, in all phases, from the pediatric realm and what can be appropriately adapted from the adult literature. • Remain mindful that approaches used in the adult population may need adaptation for the youth populations.
Preparatory assessment	• What is already known about the disease, the pediatric patient population by age group, the standard of care, and the current intervention? • What can be learned from existing literature and prior data gathered through patient partners that is relevant to this effort? Is the existing information comprehensive, representative and reliable? What is pediatric specific and what is being adapted from the adult space? • What are the gaps in the existing/known data? • What are the aims of this data collection activity? • What are the current questions to be answered in this data collection activity? • What methods will be used to solicit information? • How will the relevant paediatric community be engaged/consulted? • How will the insights gathered be documented, reviewed, and used? • How will the paediatric patient partners learn of their impact?
Outstanding questions/necessary outputs	• What needs to be learned about symptoms and disease burden, risk tolerance, expectation of benefit, other? • Are there relevant Paediatric Patient Reported Outcomes (PROs) for this patient population, and if not, what can be learned? • Are there questions about drug formulation and schedule? • What are the relevant family and caregiver considerations? • What are the outputs, including: • Key questions for paediatric patient community. • Paediatric patient engagement strategy. • Decision on paediatric patient engagement activities to conduct. • Timing of engagement. • End-to-end feedback. • What are the gaps in the study analysis plan?
Strategy	• When and how often should paediatric patients and families be approached and engaged? • What is the method(s) of engagement? Have appropriate adaptations been made for the paediatric patients? • What are the ages and maturity level of the child/adolescent; what provisions can be made to accommodate varying ages and maturities? Optimal ways to gather perspectives of younger and shyer patients must also be considered, including if, how and when to involve the adult caregiver during the youth engagement. • Does protected health information (PHI) need to be collected? If so, have the appropriate oversight bodies reviewed the plan? How will the PHI be documented, sorted, and protected? • How will data and processes be documented? • How will decisions be made re: when to conclude data collection engagement? • What policies are necessary should issues arise (data breach, non-compliance, etc.)?
Conduct	• Are Standard Operating Procedures (SOPs) in place? • What is the frequency of engagement (one time, episodic, or continuous)? • Is engagement with individual (interview, survey, or other solo engagement) or with groups (focus group discussion, other group engagement)? • Who is leading the engagement activities? Is the adult properly trained/prepared to engage with the paediatric patients? • What is the process for offering ongoing feedback in real time to paediatric participants? • Who has oversight responsibility for the engagement? • What are data capture methods? • What is the process and policy for reimbursement and compensation for paediatric patient partners and their carers?
Evaluation & feedback	• What is the data analysis plan? • Does collection of PE Data (names, mailing addresses, email addresses, account information, credit card information, zip codes, age, income and similar data) impact the study and if so, how? • What are the communication processes back to paediatric participants of, and communities involved in, engagement? • What policies guide youth who may decide to withdraw and/or who turn the age of consent during the study. • What documentation will occur for processes, challenges, and lessons learned? • Is the methodology development an iterative process?

### 2.4 Representativeness

Determining and gathering sufficiently diverse patient representation is challenging; optimal representation will depend on the disease, the population to which the study's results will be generalized, and/or the intended markets for the product or intervention. Representation of clinical trial sites may be tied to catchment area, patients served, and specific disease communities. The goals of representation should be defined in advance of patient partner outreach; diverse representation in the engagement process may enhance the likelihood of diverse trial participation. Careful attention to both demographic (e.g., age, sex, gender, race, ethnicity, socioeconomic status) and non-demographic (e.g., disease status, geographic location) factors in recruitment strategies and awareness of selection bias will impact patient experience and perspective. With respect to the continuum of age, whether the disease, response to treatment, outcome, lived experience, or other factors differ significantly across the continuum of age will inform how many paediatric partners are needed and how well one can translate findings from one age group to another.

### 2.5 Patient engagement across stakeholder groups

Meaningful engagement with paediatric advisors involves respect, a good-faith effort to consider and implement feedback, and follow-up to inform advisors of the impact of their recommendations. Patient engagement considerations vary by need, study question, stage of development, therapeutic area, and prior knowledge. Patient input at different points of a product life cycle, trial, or activity is part of a continuous engagement strategy that may deploy different, fit-for-purpose methodologies. Periodic and continuing consultation ensures that product development, trials, and the participant experience continue to meet patients’ expectations. Sponsors, clinical investigators, clinical trial sites, IRBs/ECs, and regulators should anticipate flexible and continuous paediatric involvement to meet evolving research needs with consideration of circumstance, timing, and goals. To ensure that young people's engagement and perspectives are freely offered and without other stakeholder influence, paediatric engagement through well-established channels such as Young People Advisory Groups (YPAGs) is encouraged (50).

A range of methodologies tailored for paediatric advisors may be strategically employed at multiple junctures throughout the engagement process. The sponsor or investigator may decide to conduct individual interviews, focus groups, or engage in other data collection activities at the formative stages of a study, both at the initiation of drug development program and later to validate or help interpret information, data, or results. Information from individual interviews conducted with a semi-structured interview guide may inform later activities such as focus groups; focus group data may also inform targeted areas to further explore through subsequent individual interviews. Additional activities include participant surveys or establishing advisory boards (emphasizing community, site, and family). The chosen strategy should be informed by the engagement purpose and may evolve during the product development lifecycle. Timely feedback to the paediatric advisors demonstrates respect for the time, good intentions, and value of the contribution. Contributors should know their input was thoughtfully considered, how their advice was incorporated, and, if not, why not. Importantly, if no outreach to paediatric patients and participants is planned, the justification should be provided to involved parties including ethics committees and regulatory bodies.

*IRBs and RECs* are charged with protecting the rights and welfare of participants. While IRB/REC membership might include a patient representative, it rarely (and likely never) includes a young person (although this merits serious consideration as a future goal.) Information submitted to IRBs/RECs regarding perspectives and opinions of paediatric advisors (and parents/guardians) and whether their input was incorporated can inform committee decisions. The IRB/REC may benefit from the knowledge that patients similarly situated to prospective participants have reviewed the risks and burdens of a planned protocol; that knowledge can assist in evaluating risk tolerance and risk/benefit ratio acceptability. Presentation of the patient engagement process, representation, feedback, and integration of that feedback will help the IRB/REC determine whether and how the young people's perspectives have been considered. Importantly, involvement in the design of research does not make the individual a research participant: while parental/guardian permission is required, the involvement of paediatric patient partners does not require IRB/REC review.^4^

A number of *health regulatory agencies* actively engage with patients/patent organizations to bring personal experience into scientific discussions and regulatory processes. These interactions contribute to a comprehensive picture of how health and quality of life are affected by trial design and benefit-risk considerations. Sponsors who engage with the agencies by means of any regulatory submission (e.g., a clinical trial, scientific advice, paediatric investigation plan, or marketing application) should describe patient engagement activities and their impact on trial design and program decisions with specific attention to the paediatric voice (51). In Europe, patients (but rarely young people) provide input on protocol design and paediatric investigation plans to committee discussions of benefit/risk assessment and contribute to public hearings on post-marketing pharmacovigilance measures (52). Health regulatory agencies should deploy fit-for-purpose methodology, strive for transparency and representativeness, and openly communicate the selection criteria for representative patient partners including young people themselves.

*Payers* often require evidence of a product's value, and Health Technology Assessment (HTA) bodies make decisions based on a product's cost-effectiveness; the meaning and assessment of “value” varies by geography, local or national insurance coverage, and other factors. HTA bodies consider “value” differently than regulators who assess safety and efficacy. This cost-effectiveness perspective adds economic context and complements the benefit/risk analysis typically assessed by regulators and ethics committees. These differences illustrate the benefits brought by patients to value-related judgments and determinations for reimbursement, as recognized in deliberations on potential future HTA models (53). Paediatric patients and participants and their parents/guardians should, therefore, have a prominent voice in the determination of “value.” That input informs development, study design, and outcome assessment (including PROs) and strengthens both clinical and economic value propositions for payers.

## 3 Conclusions

With select exceptions, investigators, sponsors, and other parties have not systematically gathered, shared, or incorporated the perspectives and experiences of young people in product development and clinical trials. Intentionally and explicitly elevating the voices of young people can and will impact how trials are conducted and whether study questions are responsive to the needs of this patient population. While engaging a young person is more challenging than engaging an adult, concerns can be mitigated through careful planning and communication. The additional effort necessary to engage paediatric patients and participants is integral to safe, equitable product development and to ethical trial participation by children and the adults who care for them.

## Data Availability

The original contributions presented in the study are included in the article/Supplementary Material, further inquiries can be directed to the corresponding author.

## Footnotes

^1^ https://www.icanw.org/

^2^ https://ecrin.org/projects/pedcrin

^3^ https://conect4children.org/about/

^4^Clinical trial sites and institutions should consider whether and which office or group will review the written materials (e.g., informational sheets, directions, contracts) prepared for the pediatric partner and parent/guardian. Some entities may choose the HRPP Office to perform this review, but others (e.g., Office of General Counsel) may be appropriate.
